# Investigation of anti-mullerian hormone (AMH) level and ovarian response in infertile women with endometriosis in IVF cycles

**Published:** 2018-11

**Authors:** Leili Safdarian, Seyedeh Noushin Ghalandarpoor Attar, Ashraf Aleyasin, Marzieh Aghahosseini, Fateme Sadaf Sarfjoo, Sedigheh Hosseinimousa

**Affiliations:** *Department of Obstetrics and Gynecology, Infertility Unit, Shariati Hospital, Tehran University of Medical Sciences, Tehran, Iran.*

**Keywords:** Endometriosis, Infertility, Anti-mullerian hormone

## Abstract

**Background:**

Endometriosis, can cause ovarian conflict and reduced ovarian reserve that could lead to lower response to assisted reproductive techniques

**Objective:**

Current study was conducted to determine the association between level of anti-mullerian hormone (AMH) and the infertility treatment outcomes in infertile females with endometriosis versus the non-endometriosis infertile subject.

**Materials and Methods:**

In this case-control study, 64 infertile females who referred to Shariati Hospital from April 2015 to November 2017 were enrolled. They were divided in two groups of 32 patients (endometriosis and non-endometriosis women). The anti-mullerian hormone level among all subjects was determined, treatment outcomes were evaluated and association between these factors was assessed.

**Results:**

It was seen that the anti-mullerian hormone (p=0.06), the number of retrieved oocytes (p=0.7) and embryos (p=0.7), implantation rate (p=0.6) and clinical pregnancy rate (p=0.9) were similar between two groups. In patients with stage 3 or 4 endometriosis who had lower serum AMH level significantly (p=0.001) less oocytes were retrieved (p=0.001) and less transferrable embryos (p=0.03) were achieved. However, implantation and pregnancy rates did not differ (p=0.7) (p=0.6).

**Conclusion:**

Totally, according to the obtained results, it may be concluded that ovarian reserve has more significant role in predicting infertility treatment outcome rather than receptive endometrium.

## Introduction

It seems that in patients with endometriosis, especially in severe cases because of ovarian conflict and reduced ovarian reserve, Anti-Mullerian Hormone (AMH) level could be reduced that could lead to lower response to assisted reproductive techniques (ART) (1, 2). But previous studies show conflicting results regarding the serum levels of this hormone in patients with endometriosis and response to ART (3, 4). So considering the lack of sufficient studies and conflicting results of previous studies about the level of serum AMH, ovarian reserve and ART response in patients with endometriosis, we investigated the level of AMH serum and its association with response to ART in patients with endometriosis. This could help to determine the best therapeutic approach by measuring AMH as a routine test before any therapeutic intervention to increase chances of fertility in these patients.

## Materials and methods

In this case-control study, patients were examined with idiopathic infertility or tubal factor infertility who were candidate for first IVF cycle and were referred to Shariati Hospital in 2015-2017. According to the laparoscopy treatment, before starting the cycle patients were divided into two groups; 32 patients as endometriosis and 32 patients without endometriosis as control group. Also in the endometriosis group, severity of disease was classified according to ARSM system.

It should be noted that in both groups, those with the following characteristics were excluded from the study:

1. Age over 40 yr

2. Hormonal disorders such as Cushing's disease, hypothyroidism, and hyper prolactinemia

3. Adnexal surgical history

4. Ovarian cysts in ultrasound

5. Myoma with a size of 4 cm or larger on ultrasound

AMH level was determined with ELISA method in serum samples before starting the cycle (ng/ml). Patients in both groups were divided into three subgroups based on the AMH serum levels; normal (1-5 ng/ml), lower than normal range (<1 ng/ml) and higher than normal range (>5 ng/ml). Long standard cycles with GnRH agonist (Superfact manufactured by Merck, Italy) and gonadotropins were performed in the control group. The ovarian suppression by GnRH agonist was carried out from 21^st^ day of cycles. When ovarian suppression was confirmed by ultrasound, stimulation was started by the recombinant FSH (Gonal-f manufactured by SERENO, Swiss) and HMG (Menogon manufactured by Fering, Swiss). Gonadotropin dosage was set based on age, weight and ovarian response. When at least two follicles were seen in size 18-20 mm, 10,000 IU HCG (Choragon manufactured by Fering, Swiss) was injected intramuscularly and oocytes were retrieved by trans vaginal sonography-guided, with Honda HS2600 device puncture 35 to 36 hr after HCG injection. Then fertilization was performed by In Vitro Fertilization (IVF) or Intra Cytoplasmic Sperm Injection (ICSI) technique.

It should be mentioned that initially the case group were treated with Diphereline 3.75 mg for three months every 28 days and three days after the last dose of the drug, like the control group, ovarian stimulations were started for them.

Finally the embryos, with the highest morphological degree (A and B) and if we did not have thes good quality embryos, embryos with morphological grade C or even D, were transferred to the uterus after 3 days. Patients received 400 mg vaginal micronized progesterone (Cyclogest, Actover, Britain) daily from oocyte puncture day until the day of serum βHCG testing (16 days after embryo transfer time). The measurement of βHCG in serum was performed by enzymatic antibody immunohistochemical science kit (96 tests) made in Iran with 0.5 mIU/ml sensitivity.

**Ethical consideration**

This study was approved by Ethics Committee of Tehran University of Medical Sciences [Ref. number: 92/130/300]. A written informed consent form was signed by all the participants.

**Statistical analysis**

All data were analyzed by SPSS software (Statistical Package for the Social Sciences, version 16.0, SPSS Inc., Chicago, Illinois, USA(. After collecting the required information, the frequency and percentage of qualitative variables and mean and standard deviation for quantitative variables were calculated. In this context, the Independent t-test was used to compare continuous variables and Chi-square was used to compare qualitative variables in groups. The level of significance for the interpretation of the relationships between variables in terms of number was p<0.05.

## Results

From 32 patients with endometriosis, 12 cases had endometriosis stage 1 (37.5%), 9 cases had stage 2 (28.1%), 8 patients had stage 3 (25%) and 3 cases had stage 4 (9.4%). However, in patients with endometriosis, 5 patients had less than normal AMH (15.6%), 25 patients had normal AMH (78.1%) and 2 patients had more than normal AMH (6.3%). In contrast, in the control group only one patient had less than normal AMH (3.1%), 25 patients had normal AMH (78.1%) and 5 patients had more than normal AMH (15.6%).

From 21 patients with stage 1 and 2 endometriosis, only one patient had less than normal AMH (4.7%), 18 patients had normal AMH (85.7%) and 2 patients had more than normal AMH (9.5%). From 11 patients with stage 3 and 4 endometriosis, 4 patients had less than normal AMH (36.4%), 7 patients had normal AMH (63.6%) and nobody had more than normal AMH.

In this study, although AMH level, the number of zygotes, metaphase II oocytes and obtained embryos, clinical pregnancy rates and implantation rates between the control group and the group with endometriosis had no significant difference. However, there was significant differences in AMH levels, the number of zygotes, metaphase II oocytes and obtained embryos in patients with stage 3 and 4 endometriosis compared with the control group (Table I).

Also in this study AMH level in patients with stage 3 and 4 endometriosis was significantly lower than the other two groups but the clinical pregnancies and implantation rates in these groups had no significant difference with the other groups (Table II). 

It should be mentioned that in this study, the level of AMH between the three groups in terms of fertility parameters were similar (Table II).

**Table І:** Comparison of demographic characteristics in two groups

**Characteristic**	**Stage1-2 (n=21)**	**Stage 3-4 (n= 11)**	**Endometriosis group (n= 32)**	**Control group (n= 32)**
Age (yr)	32.14	31.60	31.37	31.28
BMI (kg/m^2^)	25.30	25.24	25.27	26.13
Duration of infertility (years)	5.14	5.23	5.18	4.32

Data presented as mean.

The difference observed between groups was not significant. To analyze data we used t-test and Chi-square.

BMI: Body Mass Index

**Table II:** Comparative analysis of pregnancy outcomes in groups

**Characteristic**	**Group І**_a_ ** (stage1-2)**	**Group І**_b_ **(stage 3-4)**	**Group І (Endometriosis)**	**Group ** **II** ** (Control)**	**p-value** **(І**_a_ **-** **II** **)**	**p-value** **(І**_b_ **-II** **)**	**p-value** **(І** **-II** **)**
AMH level*	2.81	1.55	2.38	2.82	0.9	0.001	0.06
Number of oocytes**	9.43 ± 1.32	7.27 ± 1.10	8.69 ± 1.61	9.44 ± 1.60	0.7	0.001	0.7
Number of metaphase II oocytes **	6.76 ± 2.21	3.64 ± 1.91	5.69 ± 2.57	6.84 ± 2.67	0.08	0.001	0.9
Number of obtained embryos **	4.29 ± 1.30	2.45 ± 1.44	3.66 ± 1.59	4.47 ± 1.96	0.07	0.03	0.7
Implantation rate *	10.2%	9.5%	10%	12.5%	0.6	0.7	0.6
Clinical pregnancy rate *	23.8%	18.2%	21.8%	25%	0.7	0.6	0.9

To analyze data we used t-test and Chi-square.

AMH: Anti mullerian hormone

* Data presented as percentage

** Data presented as Mean ± SD

**Table III:** Compare fertility index subjects with normal AMH level in two groups

**Characteristic**	**Endometriosis group (n= 25)**	**Control group (n= 25)**	**p-value**
AMH level*	2.45 ± 0.95	2.70 ± 0.99	0.2
Number of oocytes *	8.88 ± 1.64	9.70 ± 1.68	0.08
Number of metaphase II oocytes *	6.12 ± 1.83	6.33 ± 1.68	0.6
Number of obtained embryos *	3.92 ± 1.07	4.07 ± 1.17	0.6
Implantation rate**	9.36%	9.96%	0.7
Clinical pregnancy rate**	20%	22%	0.8

To analyze data we used t-test and Chi-square.

AMH: Anti mullerian hormone

* Data presented as Mean ± SD

** Data presented as percentage

## Discussion

Based on the results of this study, inverse relationship was found between the level of AMH and severity of endometriosis. Also, fewer obtained oocytes, mature oocytes and embryos in moderate to severe endometriosis, shows a decrease in ovarian response to stimulation and lower quality oocytes in these patients compared with the control group. In other words, by increasing the severity of endometriosis, ovarian response to stimulation is reduced. In support of this, in this study when the AMH level was normal, ovarian response was not influenced by the presence or absence of endometriosis.

The main mechanism of infertility in patients with endometriosis is not fully understood. In various studies the reasons such as reduced ovarian reserve (which is caused by low levels of AMH and higher level of FSH) (5, 6) and a reduction in oocyte and embryo quality (7, 8) discussed as factors contributing to infertility in patients with endometriosis. The results of our study are generally in line with previous studies. A reduction in the level of AMH, obtained oocytes, metaphase II oocytes and transferable embryos in patients with moderate to severe endometriosis represents a negative impact of endometriosis on in vitro fertilization and fertility in these patients.

According to some studies change in endometrial receptivity indexes such as IL11, p53 and LIF and consequently reduce implantation have been proposed as one of the causes participating in infertility in patients with endometriosis (9-12). However, in this study, the implantation and clinical pregnancy was similar in the control group and patients with endometriosis (12.5% vs. 10% and 25% vs. 21.8% respectively).

Also, when the results were moderated based on AMH levels in the two groups, implantation and clinical pregnancy rate in the same AMH levels were similar in both groups (9.96% vs. 9.36% and 22% vs. 20% respectively). Similar rate of Implantation and clinical pregnancy in the present study may be due to the appropriate suppression of the pituitary before starting the stimulation cycle by GnRH agonist and stimulate by appropriate dose of gonadotropins. Previous studies support the positive role of GnRH on endometrial receptivity (13, 14).

According to the results of this study, ovarian reserve reduction is the main cause of infertility in patients with endometriosis. Reduce endometrial receptivity plays a less significant role in this concept, because after homogenization of AMH level there was no difference between the groups with and without endometriosis in ovarian response to stimulation parameters. In fact, in the same AMH level, the presence or absence of endometriosis did not impressed ovarian response and endometrial receptivity.

In this study, the inverse relationship between the level of AMH, severity of endometriosis and ovarian response to stimulation were observed as level of these hormones decreased with increasing severity of endometriosis and with reduction in level of this hormone ovarian response to stimulation is weakened.

As mentioned above, it seems that we can use AMH level as a predictive marker of ovarian response to stimulation in patients with endometriosis and through which the best approach to individual treatment can be determined to increase their chances of fertility.

This study shows that ovarian reserve has more significant role in predicting infertility treatment outcome rather than receptive endometrium. However, it should be noted that although the sample size in this study was calculated with alpha error of 5% and beta error of 20%, but given the small sample size and study it only one academic medical center, we recommend conducting a multi-center study in a broader volume to be able to present the results with greater precision and accuracy at extended range.

## Note

***This article extracted from Ph.D. Thesis. (Seyedeh Noushin Ghalandarpoor Attar)***
